# New evidence for grain specific C_4_ photosynthesis in wheat

**DOI:** 10.1038/srep31721

**Published:** 2016-08-17

**Authors:** Parimalan Rangan, Agnelo Furtado, Robert J Henry

**Affiliations:** 1 Queensland Alliance for Agriculture and Food Innovation, University of Queensland, Brisbane QLD 4072, Australia; 2 Division of Genomic Resources, ICAR-National Bureau of Plant Genetic Resources, New Delhi-110012, India

## Abstract

The C_4_ photosynthetic pathway evolved to allow efficient CO_2_ capture by plants where effective carbon supply may be limiting as in hot or dry environments, explaining the high growth rates of C_4_ plants such as maize. Important crops such as wheat and rice are C_3_ plants resulting in efforts to engineer them to use the C_4_ pathway. Here we show the presence of a C_4_ photosynthetic pathway in the developing wheat grain that is absent in the leaves. Genes specific for C_4_ photosynthesis were identified in the wheat genome and found to be preferentially expressed in the photosynthetic pericarp tissue (cross- and tube-cell layers) of the wheat caryopsis. The chloroplasts exhibit dimorphism that corresponds to chloroplasts of mesophyll- and bundle sheath-cells in leaves of classical C_4_ plants. Breeding to optimize the relative contributions of C_3_ and C_4_ photosynthesis may adapt wheat to climate change, contributing to wheat food security.

One of the key biological innovations was development of the ability of an organism to use light as the source of energy to generate chemical energy (ATP and NAD(P)H) for metabolic activities^1^ in the process commonly known as photosynthesis^2^. Evolutionarily, six phyla of prokaryotic bacteria have the ability to photosynthesize^3^, five of them using anoxygenic photosynthesis with bacteriochlorophyll and only one, the cyanobacteria, having oxygenic photosynthesis with chlorophyll^4^. Endosymbiotic associations of cyanobacteria in eukaryotes resulted in their ability to photosynthesize through chloroplasts in the process designated as “photosyntax” or “photosynthesis” in 1893 by Charles Reid Barnes^5^. Chemical energy generated from light energy is captured and used to synthesize organic compounds in higher plants in ‘dark reactions’^6^. There are many different photosynthetic pathways reported in higher plants^7^; four types *viz*., C_3_, C_4_, CAM (Crassulacean acid metabolism), and C_3_-C_4_ intermediates are widely known, while, C_4_-like (less advanced C_4_), C_3_-CAM, and C_4_-CAM intermediates have also been reported. These photosynthetic pathways, able to use CO_2_ as a carbon source, evolved in cyanobacteria around 3.5 billion years ago^8^. The key enzyme in C_3_ photosynthesis, ribulose diphosphate carboxylase (RuBisCO), was reported to have evolved around the same time as cyanobacteria^9^. The C_4_ pathway originated approximately 30 Mya (million years ago)^10^ and was first described 50 years ago^11^. The pathway provides enhanced radiation- water- and nitrogen- use efficiency^12^ especially in sub-optimal environments^10^,^13^.

Three classical C_4_ photosynthesis subtypes, NADP-ME (NADP- dependent malic enzyme), NAD-ME (NAD- dependent malic enzyme) and PEPCK (phosphoenolpyruvate carboxykinase) have been defined based upon the decarboxylation reactions involved^14^. These photosynthetic pathways explain the high growth rates of C_4_ plants such as maize. Anatomical, biochemical, and molecular evidence has been commonly used to distinguish C_4_-(sub)types from C_3_-types^15^. Kranz anatomy with reactions compartmentalized in different cell types has been considered essential for C_4_ photosynthesis^16^ but spatial compartmentalization in a single-cell has been demonstrated more recently^17^. The stem and petiole of C_3_ plants (tobacco and celery) was reported to accomplish NAD-ME type C_4_ photosynthesis in cells surrounding vascular bundles^18^. Photosynthesis in cereal grains is less well defined. Ear photosynthesis in wheat contributes from 10% to 44% of grain yield^19^. Grain photosynthesis accounts for 33-42% of this photosynthesis depending on the genotype and environment^20^.

Wheat is a major food crop critical to global food security. The current increase in wheat production of around 1% per year is not keeping pace with the rate of yield growth required to achieve the target of doubling crop production by 2050^21^. The likely impact of climate change makes progress in advancing wheat productivity more urgent. Increasing total plant biomass through efficient carbon capture by photosynthesis is now more crucial in improving wheat productivity since advances in grain yield by improving harvest index have plateaued^22^. Plants with the C_4_ pathway are known to contribute 25% of total photosynthesis although they represent just 3% of species^10^. Converting C_3_ crops to C_4_ provides the possibility of improving yield by 30% through improved water- and nitrogen- use efficiency^23^. Engineering C_3_ food crops like wheat and rice to use the C_4_ pathway has long been explored to enhance global food security^24^. We now report an analysis of the transcriptome of genes associated with C_4_ photosynthesis in the developing wheat grain. Genes identified as transcripts were located in the genome and their sequences analysed to determine likely specificity. This allowed an evaluation of substantial new evidence for C_4_ photosynthesis in wheat grains.

## Results

Remarkably, transcriptome analysis and functional annotation of genes expressed in developing wheat grains revealed the presence and expression of all genes specific to NAD-ME type C_4_-photosynthesis. When added to earlier evidence dispersed in the literature, the present discoveries suggest the functioning of a form of C_4_- photosynthesis specifically in the developing wheat grain. The transcriptome of the developing caryopsis from 35 diverse wheat genotypes (31 and 32 genotypes respectively from 14 and 30 days-post-anthesis stage with 28 genotypes in common) was analyzed by RNA-Seq. Annotation of the differentially expressed genes in the wheat grain transcriptome between 14 and 30 dpa (days-post-anthesis) indicated the presence of NAD-ME type C_4_ photosynthesis during wheat grain development. This was an unexpected finding with wheat being a well-known C_3_ crop. Wheat genes involved in C_4_ photosynthesis, the number of copies expressed in developing wheat grains and their C_4_ specificity (based on cytological and evolutionary evidence) are listed in Table 1.

### Molecular evidence

**Figure 1:**
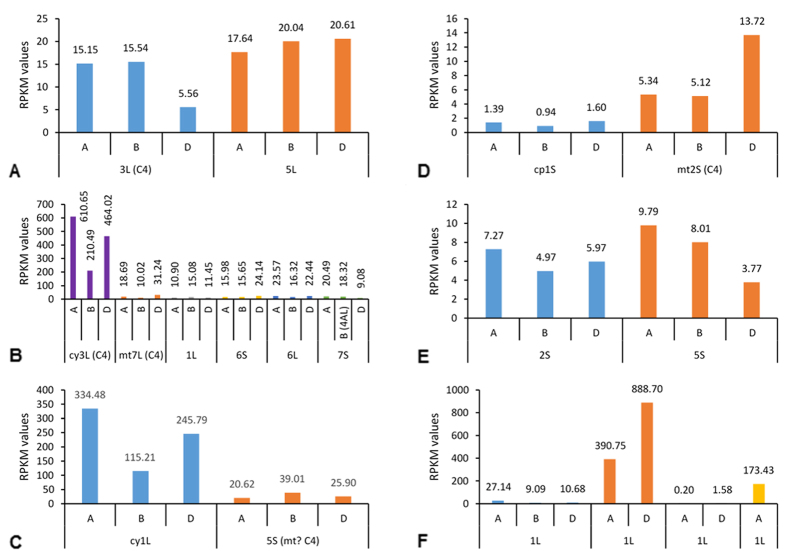
Grain specific expression of genes involved in NAD-ME type C_4_ photosynthesis as RPKM values (mean of 31 genotypes) partitioned to the sub-genome level at 14 days-post-anthesis period. (**A**) *ppc* (phospho*enol*pyruvate carboxylase); (**B)** *aat* (aspartate aminotransferase); (**C)** *mdh* (malate dehydrogenase); (**D**) *me2* (NAD-dependent malic enzyme); (**E**) *gpt* (alanine aminotransferase); (**F**) *ppdk* (orthophosphate, pyruvate dikinase). **Abbreviations used in X-axis panel–ABD:** three sub-genomes of hexaploid wheat; **cp:** chloroplast targeted; **cy:** cytosolic; **L:** chromosomal long arm; **mt:** mitochondria targeted; **S:** chromosomal short arm.

**Figure 2:**
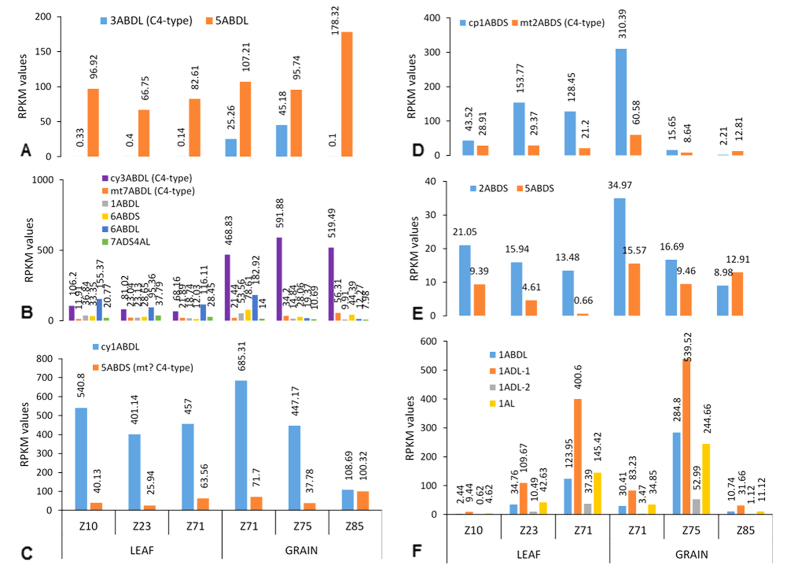
Comparison of expression levels (measured as RPKM) for genes involved in NAD-ME type C_4_ photosynthesis in leaf and grain tissues of hexaploid wheat. for X-axis, refer bottom most panel. (**A**) *ppc* (phospho*enol*pyruvate carboxylase); (**B**) *aat* (aspartate aminotransferase); (**C**) *mdh* (malate dehydrogenase); (**D**) *me2* (NAD-dependent malic enzyme); (**E**) *gpt* (alanine aminotransferase); (**F**) *ppdk* (orthophosphate, pyruvate dikinase). **Abbreviations used in the X-axis panel and series’ legends – ABD:** three sub-genomes of hexaploid wheat; **cp:** chloroplast targeted; **cy:** cytosolic; **L:** chromosomal long arm; **mt:** mitochondria targeted; **S:** chromosomal short arm; **Zadok’s scales Z10:** seedling stage; **Z23:** tillering stage; **Z71:** watery kernel stage; **Z75:** early-grain filling stage (app.14 days-post-anthesis); **Z85:** late-grain filling stage (app. 30 days-post-anthesis).

Phospho*enol*pyruvate carboxylase (*ppc*) genes were localized in wheat on the long arms of chromosomes 3 and 5. The mean expression value (in RPKM) for *ppc* across 31 genotypes at 14 dpa (chromosome 3) was 36.2 (Fig. 1A, sum of three sub-genomes A, B, and D) while only 0.29 (mean of three growth stages – Z10, Z23, and Z71 with the expression values on the Y-axis representing the sum of the three sub-genomes) for leaves^25^ (Fig. 2A), indicating a 125 fold up-regulation in the developing wheat caryopsis. Conversely, *ppc* from chromosome 5 was upregulated in leaves (Fig. 2A). It is well-known that C_4_ plants have less RubisCO protein (reflecting transcript abundance) than C_3_ plants^26^. The mean *rbcS* gene expression value was 512.3 and 39166 for the wheat caryopsis at 14 dpa and leaves respectively indicating a 76 fold down-regulation in the developing wheat caryopsis. This shows an enormous, 9500 fold, difference between developing wheat caryopsis and leaves for the relative expression of *ppc* and *rbcS* genes.

Aspartate aminotransferase (*aat*; also known as *got*) is the most up-regulated among six C_4_ pathway genes in the developing wheat caryopsis. This is also the most up-regulated gene in the leaf tissues between C_3_ and C_4_ plants^26^. Of six copies (in each sub-genome) of the *aat* gene in wheat, only two copies were the C_4_ type (cytoplasmic 3L – *aat1* and mitochondrial 7L – *aat2*). RNA-Seq analysis indicated that these genes were differentially up-regulated at 14 dpa in the developing caryopsis (Fig. 1B) when compared with leaves (Fig. 2B)^25^.

Two copies of malate dehydrogenase (*mdh*) gene were localized on the long and short arm of chromosome 1 (cytoplasmic – *mdh1*) and chromosome 5 (mitochondrial – *mdh2*) respectively across the three sub-genomes. The gene copy from chromosome 1 was differently expressed (Figs 1C and 2C) compared to the one from chromosome 5 in both grain and leaf tissues^25^. The mitochondrial targeted *mdh2* gene from chromosome 5 is likely to be involved in C_4_ photosynthesis.

Two copies of the NAD-dependent malic enzyme coding gene (*me2*) with one each targeted to chloroplast and mitochondria were localized on chromosomes 1 and 2 respectively. The mitochondrial targeted gene (chromosome 2) copy supports C_4_ photosynthesis, converting malate into pyruvate with release of CO_2_ for further fixation through the C_3_ cycle^15^. The mitochondrial isoform was up-regulated in the developing wheat caryopsis (Fig. 1D) while, the plastidic isoform was up-regulated in leaves (Fig. 2D)^25^.

Two copies of alanine transaminase (*gpt*) genes were localized to the short arm of chromosomes 2 and 5 of hexaploid wheat. This cytoplasmic enzyme converts pyruvate to alanine and vice-versa in bundle sheath and mesophyll cells respectively in a classical NAD-ME type C_4_ pathway^14^. Both genes were expressed in similar proportions in the developing wheat caryopsis at 14 dpa (Figs 1E and 2E); while the gene on chromosome 2 was more highly expressed in leaves^25^.

Pyruvate, orthophosphate dikinase (*ppdk*) gene was localized to the long arm of chromosome 1 in hexaploid wheat. All four gene copies (although a full length sequence was not available) were used to assess the RPKM expression levels in the developing wheat caryopsis at 14 dpa (Fig. 1F) and in leaf (Fig. 2F) tissues^25^. Earlier reports indicate the role of a dual promoter in regulating a single gene copy during light and dark in the chloroplast and cytoplasm respectively with the second promoter region in the first intron for cytoplasmic expression^27^. Aoyagi and co-workers showed the presence of PPDK and RubisCO in the green pericarp, but failed to envision the possibility of C_4_ photosynthesis due to the lack of Kranz anatomy in developing wheat grains^28^.

Six genes (excluding carbonic anhydrase) were involved in the NAD-ME type C_4_ pathway, phospho*enol*pyruvate (PEP) carboxylase (*ppc*), aspartate aminotransferase (*aat*; also known as *got*), malate dehydrogenase (*mdh*), NAD- dependent malic enzyme (*me2*), alanine aminotransferase (*gpt*), and pyruvate, orthophosphate dikinase (*ppdk*)^15^. Grain specific expression of genes involving NAD-ME type C_4_ photosynthesis *viz*., *ppc*, *aat*, *mdh*, *me2*, *gpt*, and *ppdk*; in all three (A, B, and D) sub-genomes (Fig. 1) indicates a possible evolutionary diversification point well before the speciation of the diploid progenitors in the Triticeae tribe. Endosperm and aleurone transcripts^29^ do not express all of these genes demonstrating that the C_4_ pathway is restricted to the wheat pericarp.

### Varied expression pattern between wheat genotypes

**Figure 3:**
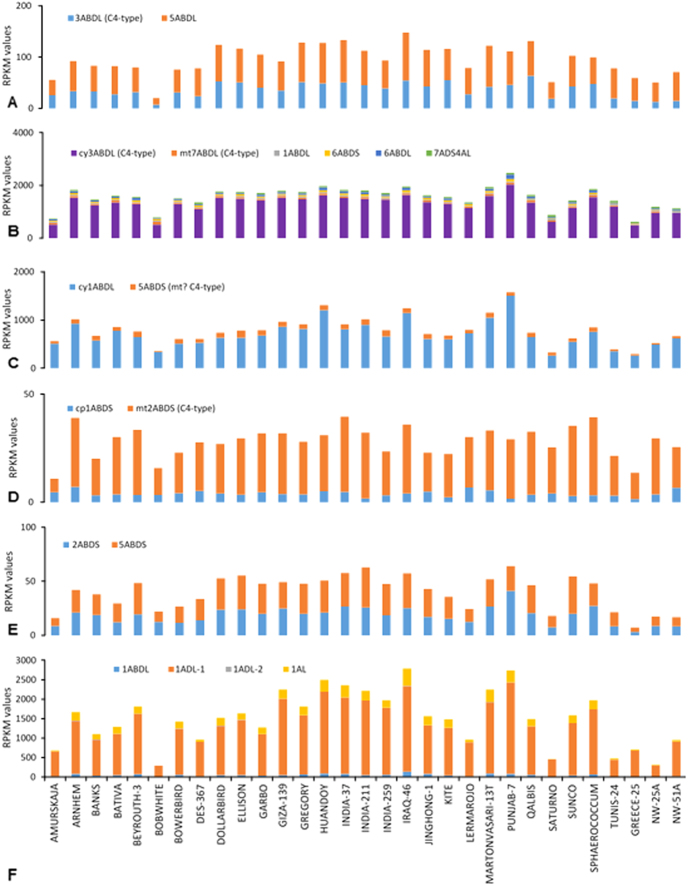
Variation in expression levels (measured as RPKM) for genes involved in NAD-ME type C_4_ photosynthesis in 31 wheat genotypes at 14 days-post-anthesis. For X-axis, refer ‘panel F’. (**A**) *ppc* (phospho*enol*pyruvate carboxylase); (**B**) *aat* (aspartate aminotransferase); (**C**) *mdh* (malate dehydrogenase); (**D**) *me2* (NAD-dependent malic enzyme); (**E**) *gpt* (alanine aminotransferase); (**F**) *ppdk* (orthophosphate, pyruvate dikinase). **Abbreviations used in series’ legends – ABD:** three sub-genomes of hexaploid wheat; **cp:** chloroplast targeted; **cy:** cytosolic; **L:** chromosomal long arm; **mt:** mitochondria targeted; **S:** chromosomal short arm.

The presence of all C_4_ specific genes in the genome confirms that natural selection may have already explored the options being considered by plant breeders^30^. The levels of expression for all six genes at 14 dpa in NAD-ME type C_4_ pathway varied across 31 genotypes (Fig. 3) suggesting potential for genetic selection for this trait in wheat breeding.

### C_4_ specificity of gene sequences

Four of the six genes involved in NAD-ME type C_4_ photosynthesis, (*aat*, *mdh*, *me2*, and *ppdk*) had sub-cellular targeting that suggests C_4_-type specificity^15^. The other two genes (*ppc* and *gpt*) require sequence information to distinguish between the copies specific for C_3_- or C_4_- pathways. Analysis of *gpt* genes in wheat suggested both C_3_ and C_4_ forms were expressed at similar levels (Figs 1E and 2E) across photosynthetic and non-photosynthetic tissues. While the *ppc* gene copies clearly show different expression patterns between developing grains and leaves (Figs 1A and 2A); sequence differences are the only way to distinguish the C_3_- and C_4_- isoforms. Specific amino acid substitutions have been associated with C_4_ functionality^13^. Increased tolerance to feedback inhibition by malate involves **G**_**884**_ (Glycine) in C_4_-isoforms rather than **R**_**884**_ (Arginine) as found in C_3_-isoforms. The translated sequence of the *ppc* gene from chromosome 3 (**S**_**885**_) and 5 (**R**_**891**_) of wheat cDNA (IWGSC – international wheat genome sequencing consortium, release-23 version) indicates the gene copy from chromosome 5 is C_3_-type; while the chromosome 3 copy is non-C_3_ type. The gene sequences from wheat and related species^31^ were analyzed using the translated amino acid sequence of the *ppc* gene (IWGSC cDNA database release-23) from chromosome 3. Results indicated that most of the Triticeae tribe members have five copies of *ppc* gene (Table 2) although in the hexaploid wheat cDNA database we found only two copies (3L and 5L). IWGSC cDNA database (release-23)^31^ was used to perform tblastn analysis with the translated *ppc* gene sequence confirming that gene sequence copies from chromosomes 3S, 6 and 7 are not in frame suggesting the presence of insertions or deletions in these genes. However, one *ppc* gene copy from all Triticeae members had **S**_**885**_indicating a non C_3_-type; while the other four copies revealed a C_3_-type – **R**_**891**_ (the corresponding amino acid position) across all the Triticeae members studied (Table 2). Since the amino acid position is neither **R** nor **G**, we studied different species acting as diversification points in the evolution of these species in order to compare them with respect to known C_4_ types (Table 3). This gave an indication that from Bryophytes to Angiosperms, the C_3_ type amino acid position was invariably conserved with **‘R’** (Table 3). Whereas the C_4_ type amino acid position was either **S** (*Panicum* and Triticeae tribe) or **Q** (*Alloteropsis*, *Setaria*) or **G** (*Alloteropsis*, *Panicum*, *Zea*, and *Sorghum*) or **I** (*Amaranthus*) depending on the species or taxonomic group (Table 3).

## Discussion

Wheat is widely known as a classical C_3_ plant. Close examination of the literature shows many reports of components of the case for C_4_ photosynthesis in the grain especially in early studies. However, this evidence has been overlooked because of the knowledge of C_3_ photosynthesis in the leaves and a lack of understanding of the possibility of different pathways in different parts of the plant. Indeed many studies have attempted to explain away the evidence that did not fit with the knowledge that wheat was a C_3_ plant. This study has identified a complete set of C_4_ specific genes in wheat genome for the first time. This finding addresses the apparent anomaly of this subfamily (Pooideae) of the Poaceae being uniquely seen to lack C_4_ photosynthesis. We have also shown for the first time that all the required genes are expressed in the required compartmentalization, specifically in the pericarp, a tissue with an anatomy that is suitable for supporting a C_4_ pathway. The possibility of photosynthesis in the pericarp of wheat grains was predicted in the early 1960s^32^. Phospho*enol*pyruvate carboxylase (PPC) from the wheat or barley pericarp tissues of developing grain was reported to be 50-100 times as active in carbon fixation as ribulose diphosphate carboxylase (RuBisCO)^33^. Based on the enzyme activity for malate dehydrogenase, malic enzyme, and pyruvate-orthophosphate dikinase in pericarp tissues of developing grain, Duffus and Rosie^33^ indicated the possibility of C_4_ photosynthesis. A little later, Wirth, *et al*.^34^ studied different reproductive parts from wheat and oat – glume, lemma, palea, and pericarp – along with leaves and reported that the pericarp tissues of developing grains seemed to “possess carbon metabolism different to that of the other tissues”. They also analyzed and reported the possibility of refixation of the CO_2_ released through respiration or photorespiration. Assimilation of ^14^CO_2_ to malate and 3-phosphoglyceric acid in wheat ears and flag leaf respectively; along with higher enzyme activities for enzymes of C_4_ and C_3_ metabolic pathways in ears and flag leaf respectively suggested the possibility of C_4_ photosynthesis in ears^35^. Carbon isotope discrimination (Δ) values were used to distinguish plants between C_3_- and C_4_-type^36^. Although wheat was considered a C_3_ plant, Δ values were used to study the plants’ water-use- or transpiration efficiency^37^,^38^. Their results indicate a clear difference between flag leaf and grain Δ values in different wheat genotypes. Although the difference is not as distinct as it is with classical C_4_ photosynthesis. This might be due to either inefficient less advanced C_4_ type photosynthesis or the fact that grain photosynthesis accounts for only 33-42% of ear photosynthesis^20^ with the remainder translocated from leaf or stem tissues with C_3_-type photosynthesis thereby diminishing the difference in Δ values between flag leaf and grain to a marginal level. Similarly but in reverse, in a maize plant with C_4_-type, maize husk leaves were reported to be C_3_-type and their Δ values were marginally higher than leaves^39^.

In spite of this evidence (enzyme activity, ^14^CO_2_ in malate, Δ values), earlier researchers failed to explore C_4_ photosynthesis in wheat grains due to the view that Kranz anatomy was required for C_4_ photosynthesis^16^,^28^. In 2001 and 2002, the occurrence of C_4_ photosynthesis without Kranz anatomy was reported in single cells and in the petioles of C_3_ plants respectively^17^,^18^.

In the late 1990s, there were reports of the C_4_ pathway being found selectively at different developmental stages of some plants (*Salsola* spp. and *Haloxylon* spp.) of cotyledons and leaves exhibiting C_3_ and C_4_ type photosynthesis respectively^40^,^41^. Similarly there have been reports on the selective use of the C_4_ pathway in different environments like terrestrial or submerged situations^42^,^43^ or high or low CO_2_^44^. Selective expression of the C_3_ pathway was reported in the husk leaves (hypsophylls) of the maize plant^45^ which is otherwise a C_4_ plant. Evidence of 4-carbon compounds specifically in wheat leaf bases^46^ agreed with a much later report of C_4_ pathways in C_3_ plants^18^. Altered C_4_ and C_3_ enzymatic activity has recently been reported in wheat ears under water stress^47^ but the significance of this was not clear given the C_3_ status of wheat. This evidence suggests the presence of a diversified range of regulatory patterns of C_4_ pathway in plants with ontogeny and varied environmental cues. Operation of different pathways (C_3_ or C_4_) at different growth stages allows wheat to have a lifecycle that extends across seasons with varying environments (cool, wet during vegetative growth; hot, dry during grain filling).

### Molecular and cytological evidence

Functional annotation and differential expression of C_4_-specific gene copies (Figs 1 and 2) for genes of NAD-ME type photosynthesis specifically in developing wheat grains adds evidence for the C_4_ pathway in wheat grains as suggested in early reports^34^,^35^. With multiple copies in a genome, species preferentially co-opt the same neo-functionalized gene lineage for C_4_ photosynthesis^48^ although these genes were present well before the evolution of the C_4_ pathway but with different anaplerotic functions^49^.

**Figure 4:**
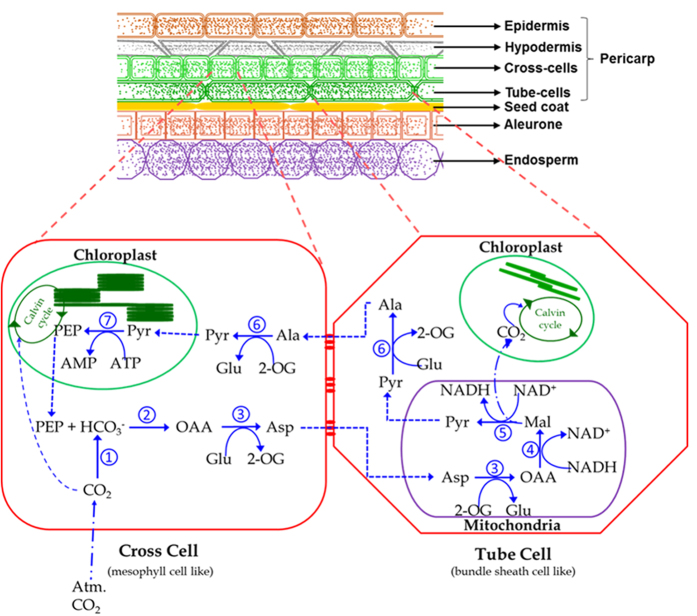
Schema depicting a longitudinal cross section of developing wheat grains with one cross- and tube-cell in enlarged view illustrating the C_4_-like type photosynthetic pathway through NAD-dependent malic enzyme (NAD-ME) subtype [adapted and modified from Sage and Monson^15^]. **2-OG:** 2-Oxyglutarate; **Ala:** Alanine; **Asp:** aspartate; **Glu:** glutamate; **Mal:** malate; **OAA:** oxaloacetate; **PEP:** phospho*enol*pyruvate; **Pyr:** pyruvate; **1,** Carbonic anhydrase (CA, EC 4.2.1.1); **2,** PEP carboxylase (PPC, EC 4.1.1.31); **3,** Aspartate aminotransferase (AAT, EC 2.6.1.1); **4,** Malate dehydrogenase (MDH, EC 1.1.1.31); **5,** NAD-dependent-malic enzyme (ME2, 1.1.1.39); **6,** Alanine aminotransferase (GPT, EC 2.6.1.2); **7,** Pyruvate orthophosphate dikinase (PPDK, EC 2.7.9.1).

Reports indicate that cross- and tube-cells in pericarp of developing wheat grain are photosynthetic in nature and contribute to the grain weight^50^. Thorough re-examination of this report indicates the presence of numerous mitochondria, and dimorphic chloroplasts – stacked grana in cross-cells and reduced stacking in tube-cells being structurally similar to classical C_4_ types^51^. The presence of numerous mitochondria specifically in bundle sheath cells of NAD-ME type C_4_ pathway has also been reported^52^. These pieces of cytological evidence in addition to our molecular evidence suggests NAD-ME type C_4_ photosynthesis operates in developing wheat grains (Fig. 4) with cross- and tube-cells paralleling mesophyll and bundle sheath cells in a classical C_4_ pathway. In contrast to the classical C_4_ photosynthesis that is associated with little or no starch granules in mesophyll cells^53^, the presence of starch granules in cross-cells (mesophyll like) was reported by Morrison^50^. This led us to question the possibility of NAD-ME type C_4_ photosynthesis in wheat grains. However, there is evidence for the presence of RuBisCO in both mesophyll and bundle sheath cells of young amaranth leaves^54^ suggesting a C_3_ cycle in both mesophyll and bundle sheath cells. In some *Flaveria* spp., reports of the presence of RuBisCO in both mesophyll and bundle sheath cells supporting both the C_3_ and C_4_ cycle simultaneously led to their classification as having C_4_-like type (less advanced) photosynthesis^7^. Occurrence of the C_3_ cycle (presence of RuBisCO) in both mesophyll and bundle sheath cells along with the C_4_ pathway in some species might be due to the fact that compartmentalization of RuBisCO is the final step in the evolution of C_4_ from C_3_ photosynthesis^55^. These considerations lead us to propose the occurrence of C_4_-like type (less advanced) photosynthesis in developing wheat grains through the cross- and tube-cell layers of pericarp paralleling the mesophyll and bundle sheath cells of classical C_4_ photosynthesis (Fig. 4).

### Taxonomical and evolutionary evidence

Around 41% of grasses are known to fix carbon through the C_4_ pathway^56^. Hence, an overview at evolutionary scale linking speciation events with C_4_ photosynthesis might shed light on the evolution of the C_4_-like type photosynthetic pathway in developing wheat grains. The Poaceae family is monophyletic and consists of 12 subfamilies with three at the basal level, followed by the BOP and PACMAD clades consisting of three and six subfamilies each with the Triticeae tribe included in the subfamily Pooideae (cool season grasses) of the BOP clade^56^. To date, no species from the Pooideae have been reported to be C_4_. The Aristidoideae (PACMAD) subfamily has been reported to have at least two independent evolutions of the C_4_ pathway^56^. In this study, knowledge of specific amino acids (**G** in C_4_ and **R** in C_3_) in the *ppc* gene product required for efficient carbon fixation by the C_4_ pathway^13^ was used to show that wheat and all related species (including *Hordeum* and *Brachypodium*) had five *ppc* copies in their genome with four of them having the amino acid **R** indicating their C_3_ nature while one copy (3L, as in hexaploid wheat) has an **S** – a non-C_3_ type in place of **R** except *Brachypodium* (Table 2).

The C_3_ specific amino acid position (**R**) was apparently conserved (Table 3) from Bryophytes (around 450Mya) to Angiosperms. The amino acid position with C_4_ specificity appears to have evolved at least four times in the last 30Mya (origin of C_4_) with either **S** (*Panicum* and Triticeae tribe) or **Q** (*Alloteropsis*, *Setaria*) or **G** (*Alloteropsis*, *Panicum*, *Zea*, and *Sorghum*) or **I** (*Amaranthus*). This suggest that various amino acid substitutions at that site might result in differing efficiency of carbon fixation through the C_4_ pathway by altering tolerance to feedback inhibition by malate^13^. Analysis of enzyme kinetics with each of the four C_4_-specific amino acids individually might help to rank their photosynthetic efficiency. However, the weakest form among the four will probably be much more efficient in carbon fixation than the C_3_ type (**R**). The presence in wheat of an amino acid specific to a known C_4_-type (**S** in *Panicum laetum* and *P. miliaceum*) is strong evidence when taken together with the grain specific pattern of expression of the C_4_ specific *ppc* gene (Fig. 2A). The tribe Brachypodieae (*Brachypodium distachyon*) has amino acids corresponding to the C_3_-type for all the five copies; while members from tribe Triticeae (*Aegilops*, *Hordeum*, *Triticum*) have one copy of the C_4_-type and four copies of the C_3_-type (Table 2). This fits with the evolutionary time line for C_4_ photosynthesis around 30Mya^10^; with Brachypodieae evolving around 35Mya^57^ having only C_3_-type genes (*Brachypodium*). Unfortunately, there are no diversification points between *Brachypodium* (35 Mya) and *Hordeum* (11.6 Mya)^57^ to establish the exact timing of C_4_ evolution in the Pooideae tribe. Derived traits like those associated with C_4_ photosynthesis appear at later evolutionary stages and are expressed later in plant development^58^. This is consistent with the observations of C_4_ photosynthesis in wheat and its relatives specifically in the grain.

Based on this molecular, cytological, taxonomical and evolutionary evidence, we propose the occurrence of C_4_-like type photosynthesis specifically in developing wheat grains. Recognition of both C_3_ and C_4_-like type photosynthetic pathways in wheat provides a basis for interpretation of wheat performance as a crop adapted to maturation in hot dry environments, suggesting that the plant may rely more on C_4_ photosynthesis under conditions of water stress during the grain filling stage. Photosynthates from pericarp, glumes and awns are critical^59^ when other parts of the plant lose photosynthetic capacity due to terminal drought often experienced in the environments in which wheat evolved. This may be especially important in the development of wheat varieties to adapt to climate change^60^ and associated temperature extremes. The operation of C_4_ photosynthesis specifically in these tissues provides an adaptive advantage to the wheat plant while C_3_ photosynthesis is adequate during early vegetative growth under more temperate conditions. The potential for genetic manipulation to extend C_4_ photosynthesis throughout the wheat plant seems much more realistic given the existing expression of the entire pathway in the grain. This supports the view that plant species have evolved specific photosynthetic pathways in different organs, at specific developmental stages and in different environments suggesting that the classification of plants as C_3_ or C_4_ or CAM in a broad fashion cannot simply be based upon leaf anatomy. Research to establish the variation in flux through this pathway in wheat and its progenitors will shed much light on the share of carbon fixation through the C_3_ and C_4_ pathway under varying environmental conditions. This has the potential to suggest new options for the development of higher yielding wheat genotypes.

### Experimental procedures

#### Experimental material

Thirty-five wheat genotypes *viz*., Amurskaja, Arnhem, Banks, Bativa, Beyrouth-3, Bobwihte-26, Bowerbird, Des-367, Dollarbird, Ellison, Garbo, Giza-139, Gregory, Huandoy, India-37, India-211, India-259, Iraq-46, JingHong-1, Kite, LermaRojo, Martonvasari-13T, Punjab-7, Qalbis, Saturno, Sunco, Sphaerococcum, Tunis-24, Greece-25, NW-25A, NW-51A, NW-93A, NW-108A, Pelada, and Vega were used for transcriptome analysis. Seeds for these genotypes were procured from the Australian Winter Cereals Collection. Seeds were germinated and plants grown under controlled conditions as described in Furtado, *et al*.^58^. Developing grains were collected from wheat spikes at 14 days- and at 30 days-postanthesis (dpa) as described elsewhere^58^.

#### RNA isolation, library preparation and NGS sequencing

RNA isolation, cDNA synthesis, library preparation and next generation sequencing was carried out and described by Furtado, *et al*.^58^. Libraries for 31 samples from 14 dpa and 32 samples from 30 dpa with 28 genotypes in common were prepared and sequenced as described in Furtado, *et al*.^58^. Libraries were not prepared for four cultivars *viz*., NW-93A, NW-108A, Pelada, and Vega at 14 dpa, and three cultivars *viz*., Greece-25, NW-25A, and NW-51A at the 30 dpa stage due to lack of sufficient starting material.

#### Sequencing data processing and analysis

Sequencing data obtained was imported into CLC genomics workbench ver. 7.0.4 (CLC Bio-Qiagen, Denmark) and further processing and analysis were done within this environment unless otherwise stated. Quality checking, trimming, and RNA-Seq analysis were performed as described in Furtado, *et al*.^58^ using the TaGI (*Triticum aestivum* Gene Indices, The Computational Biology and Functional Genomics Laboratory, Dana Farber Cancer Institute and Harvard School of Public Health) cDNA database as reference, containing 221,925 sequences (release 12.0)^61^.

#### Differential transcript and statistical analyses

Transcripts that were differentially expressed between 14 and 30 dpa were analyzed using the RNA-Seq experimentation tool with default parameters. Statistically significantly differentially expressed transcripts were identified using both Gaussian (mean based) and Empirical analysis of Differential Gene Expression (EDGE, count based) statistics facilitated through CLC workbench (CLC Bio-Qiagen, Denmark) with *p*-value using false discovery rate (FDR) corrected least the significant difference set at 0.01 level.

#### Functional annotation and data mining

In total, 26,477 transcripts that are common for both Gaussian and EDGE statistics were significant at FDR corrected value 0.01. Among them, 319 and 181 transcripts were unique to 14 dpa and 30 dpa respectively; while 16237 and 9740 transcripts were differentially up-regulated at 14 dpa and 30 dpa respectively. Transcript sequences for these four groups (unique 14 dpa, unique 30 dpa, differential 14 dpa and differential 30 dpa) were extracted from the reference database (TaGI) and subjected to blastx analysis against the non-redundant protein database. Blast results obtained were converted to a BLAST2GO project file and exported in “.dat” format files using the plug-in version within CLC workbench (CLC Bio-Qiagen, Denmark). Functional annotation for these four groups was performed independently using BLAST2GO Pro ver 3.0.10 with default parameters^62^. Annotations were augmented using InterProScan and followed by Run-annex options. Annotations pertaining to the plant database were retained using the GO (gene ontology)-slim option. Finally, KEGG (Kyoto encyclopedia of genes and genomes) pathway maps for these four annotated sequence groups were retrieved using GO-enzyme code mapping option. The differential 14 dpa group highlighted the presence of a complete C_4_ photosynthetic pathway existing in developing wheat caryopsis.

#### Chromosomal localization and IWGSC transcript retrieval

Based on enzyme code mapping, TaGI transcript IDs pertaining to those enzyme code (EC numbers) for the genes involved in the C_4_ photosynthetic pathway from the differential 14 dpa group were retrieved using CLC workbench (CLC Bio-Qiagen, Denmark). A total of 62 transcripts for the six genes (phosphoenolpyruvate carboxylase – *ppc*; aspartate aminotransferase – *aat*; malate dehydrogenase – *mdh*; decarboxylating dehydrogenase – *me2*; alanine aminotransferase – *gpt*; and pyruvate, orthophosphate dikinase - *ppdk*) were retrieved. Blast searches for the 62 transcripts from the TaGI database were performed against the IWGSC cDNA database containing 100,717 sequences (release-23)^31^ for retrieval of IWGSC transcripts (since the TaGI transcripts are lesser in length and mostly incomplete).

#### Modified reference and RNA-Seq analysis

Based on blast analyses using the 62 transcripts of TaGI as reference^55^ transcripts from the IWGSC cDNA database (release-23)^31^ were obtained. Using sub-genome sequence information and sequence alignment, 10 of 55 transcripts were found to be actually five genes each being two parts of the same transcript with or without overlap. Based on homology and sequence alignment between the sub-genome copies, those 10 transcripts were stitched into five transcripts resulting into a total of 50 transcripts for six genes that accomplish NAD-ME type C_4_ photosynthesis. In order to construct a modified reference, 10 transcripts (that are used to stitch) were replaced with the five stitched transcripts in the 100,717 sequences of the IWGSC cDNA (release-23)^31^. The resulting database containing 100,712 sequences was named “modified IWGSC cDNA (release-23)”and used for performing RNA-Seq analysis as described above^58^ to obtain RPKM values for the 31 genotypes at the 14 dpa stage. Although researchers use FPKM instead of RPKM for paired-end reads, we used RPKM with an option of counting mapped paired-end reads as “two” and singleton reads that are mapped as “one” to avoid confusion between FPKM and RPKM terminologies.

#### RNA-Seq analysis for tissue specific transcriptome data

Raw reads (100 bp paired-end sequencing on Illumina HiSeq2000) of different tissues (leaf, and grain) at three different growth stages for hexaploid wheat (‘Chinese Spring’) were available online^25^. These raw sequence reads were downloaded, and processed through the CLC workbench (CLC Bio-Qiagen, Denmark). Quality checking, trimming and RNA-Seq analysis using the modified IWGSC cDNA (release-23) containing 100,712 sequences as reference were performed to obtain RPKM values and represented in pictorial form.

#### Taxonomical and evolutionary relation for C_4_-specificity

Specific amino-acid positions for PPC (PEPCase) that are functionally related to C_3_ and C_4_-specificity were reported recently^13^. In order to identify these in wheat and related species (Table 2), whole genome sequence details^63^ were downloaded and translational blast analysis was performed using CLC workbench ver. 8.5.1 (CLC Bio-Qiagen, Denmark).

Similar analyses were performed for species (for which genome sequence was available) including taxa from bryophytes to angiosperms^31^,^64^,^65^ corresponding to various diversification points in an evolutionary timeline (Table 3). Although whole genome data for some well-known C_4_ species was not available, *ppc* gene sequences in public databases was used to study the evolutionary pattern at specific amino acid positions (Table 3) that are functionally related to C_3_ or C_4_ specificity.

## Additional Information

**How to cite this article**: Rangan, P. *et al*. New evidence for grain specific C_4_ photosynthesis in wheat. *Sci. Rep.* **6**, 31721; doi: 10.1038/srep31721 (2016).

## Figures and Tables

**Table 1:** Wheat genes identified as being involved in NAD-ME type C_4_ photosynthesis, chromosomal localization, and C_3_-, C_4_-type specificity.

Gene name	Copy number	C_4_-type^	C_3_-type^
*ppc*	2	3ABDL	5ABDL
*aat*	6	3ABDL (cyt) – *aat1*	1ABDL
		7ABDL (mt) – *aat2*	6ABDL
			6ABDS
			7ADS4AL
*mdh*	2	5ABDS (mt) – *mdh2*	1ABDL (cyt) – *mdh1*
*me2*	2	2ABDS (mt)	1ABDS (plastid)
*gpt*	2	2ABDS & 5ABDS (both cyt)	
*ppdk*	4	1ABDL (cp)	1ABDL (cyt)

^A, B and D represent the there sub-genomes in hexaploid wheat; ‘L’ and ‘S’ represent the long and short chromosomal arms; ***aat***: aspartate aminotransferase (also known as *got*); **cp**: chloropolastic; **cyt**: cytoplasmic; ***gpt***: alanine aminotransferase; ***mdh***: malate dehydrogenase; ***me2***: NAD-dependent malic enzyme; **mt**: mitochondrial;***ppc***: phospho*enol*pyruvate carboxylase; ***ppdk***: pyruvate, orthophosphate dikinase..

**Table 2:** Amino acid (10^th^ position, bold) for C_4_ specificity across species that are taxonomically related to *Triticum* spp.

Species^a^	Amino acid with flanking region	Chr no	Type	Reference
*Brachypodium distachyon*	LEGDPYLKQRLRLRDPY	1	C_3_	Kersey, *et al*.^31^
	LESDPYLRQRLMLRDSY	2	C_3_	
	LEGDPYLRQRLRLRE??	2	C_3_	
	LEGDPYLKQRLRLRESY	3	C_3_	
	LEGDPYLKQRLRLRDAY	4	C_3_	
*Hordeum vulgare*	LESDPYLRQRLMLRDSY	3	C_3_	Kersey, *et al*.^31^
	LEGDPYLRQ**S**LRLRDSY	3	**C**_**4**_**?**	
	LEGDPYLKQRLRLRDAY	5	C_3_	
	LEGDPYLKQRLRLRESY	6	C_3_	
	LEDDPYLKQRLRLRDPY	7	C_3_	
*A. sha, spe, tau; T. mon, ura; T. durum* (cappelli)	LESDPYLRQRLLLRDSY	3?	C_3_	IWGSC^63^
	LEGDPYLRQ**S**LRLRDSY	3?	**C**_**4**_**?**	
	LEGDPYLKQRLRLRDAY	5?	C_3_	
	LEGDPYLKQRLRLRESY	6?	C_3_	
	LEDDPYLKQRLRLRDPY	7?	C_3_	
*T. durum* (strongfield)	LESDPYLRQRLLLRDSY	3?	C_3_	IWGSC^63^
	LEGDPYLRQ**S**LRLRDSY	3?	**C**_**4**_**?**	
	LEGDPYLKQRLRLRESY	6?	C_3_	
	LEDDPYLKQRLRLRDPY	7?	C_3_	
*T. aestivum*	LESDPYLRQRLLLRDSY	3S	C_3_	Mayer, *et al*.^65^
	LEGDPYLRQ**S**LRLRDSY	3L	**C**_**4**_**?**	
	LEGDPYLKQRLRLRDAY	5L	C_3_	
	LEGDPYLKQRLRLRESY	6ASBDL	C_3_	
	LEDDPYLK?????????	7DL		
	LEGDPYLRQRLQLRDPY	4DS	C_3_	

^a^**A. sha:** Aegilops sharonensis; **spe:** A. speltoides; **tau:** A. tauschii; **T. mon:** Triticum monococcum; **ura:** T. uratu;

**Table 3:** Amino acid (10^th^ position, bold) for C_4_ specificity across species with different diversification point in evolutionary scale (in Mya).

Species^a^	Amino acid with flanking region	Type	Taxonomy	Mya	Reference
*Physcomitrella patens*	LQGNPTLKQRLRLREPY	C_3_	Bryophytes	450	Rensing, *et al*.^66^
	LQGNPSLKQRLRLREPY	C_3_			
	LQGNPTLKQRLRLREPV	C_3_			
	LEGNPTLKQRLRLREQY	C_3_			
*Selaginella moellendorffii*	LAGNPILKQRLSLREPF	C_3_	Lycophytes	410	Banks, *et al*.^67^
	LEENPTLKQRLRLREPF	C_3_			
PA, AS, GG, JC, PS, TB	LEGDPYLKQRLRLRDSY	C_3_	Gymnosperm	300	Nystedt, *et al*.^64^
*Amborella trichopoda*	LEGDPYLKQRLRLRDSY	C_3_	Basal Angiosperm	130	Soltis, *et al*.^68^
*Oryza sativa*	LEGDLYLKQRLRLRNAY	C_3_	Oryzoideae (BOP clade)^p^	60^q^	Kersey, *et al*.^31^
	LEGDPYLRQRLRIRDSY	C_3_			
	LEGDPYLKQRLRLRDAY	C_3_			
	LEGDPYLKQRLRLRESY	C_3_			
*Guadua* sp.	LEGDPYLKQRLRLRESY	C_3_	Bambusoideae (BOP clade)^p^		Christin, *et al*.^69^
	LESDPYLRQRLMLRDSY	C_3_			
*Brachypodium*	Refer Table 2	C_3_	Pooideae (BOP clade)^p^	35^q^	
Triticeae	Refer Table 2	?		11.6^q^	
*Alloteropsis* spp.	LEGDPYLKE**G**LRLRNPY	C_4_^b^	Panicoideae (PACMAD clade)^p^	20-27^q^	Christin, *et al*.^69^
	LEGDPYLKQ**Q**LRLRDPY	C_4_^c^			
	LEGSPGLKQRLRLRDPY	C_3_^d^			
	LEGDPYLKQRLRLRESY	C_3_^e^			
	LEGDPYLKQRLRIRDSY	C_3_^f^			
	LEGDPYLKQRLRLRDAY	C_3_^g^			
	LEGGPYLKQRLRLRDPY	C_3_^h^			
	LEGDLYLKQRLRLRDAY	C_3_^i^			
	LEGDPYLKQRLRLRDPY	C_3_^j^			
*Panicum* spp.	LEGDPFLKQ**S**LRLRNPY	C_4_^k^	Panicoideae (PACMAD clade)^p^	18-27^q^	Christin, *et al*.^70^ Christin, *et al*.^69^
	LEGDPYLKQ**G**LRLRNPY	C_4_^l^			
	LEADPFLKQ**S**LRLRNPY	C_4_^m^			
	LEGDLYLKQRLRLRDAY	C_3_^n^			
	LEGDPYLKQRLRLRDPY	C_3_^o^			
*Setaria italica*	LESDPGLKQ**Q**LRLRDPY	C_4_	Panicoideae (PACMAD)^p^	16-27^q^	Bennetzen, *et al*.^71^ Christin, *et al*.^69^
	LEGDPYLKQRLRLRESY	C_3_			
	LESDPGLQQ**Q**LMLRDSY	C_3_			
	LEGDLYLKQRLRLRDAY	C_3_			
	LEGDPYLKQRLRIRDSY	C_3_			
*Zea mays*	LEGDPFLKQ**G**LVLRNPY	C_4_	Panicoideae (PACMAD)^p^	16^q^	Schnable, *et al*.^72^
	LEGDLYLKQRLRLRDAY	C_3_			
	LEGDPYLKQRLRIRDSY	C_3_			
	LEGDPYLKQRLRLRESY	C_3_			
*Sorghum bicolor*	LEGDPYLKQ**G**LRLRNPY	C_4_	Panicoideae (PACMAD)^p^	13^q^	Paterson, *et al*.^73^
	LEGDLYLKQRLRLRDAY	C_3_			
	LEGDPYLKQRLRIRDSY	C_3_			
	LEGDPYLKQRLRLRESY	C_3_			
	LEGDPYLKQRLRLRDAY	C_3_			
*Amaranthus hypochondriacus*	LDADPYLKQ**I**LRLRDPY	C_4_	Dicot	-	ADO15315 (Accession number)

^a^Species arranged in the order of evolution except Amaranthus**; PA:** Picea abies; **AS:** Abies sibiria; **GG:** Gnetum gnemon; **JC:** Juniperus communis; **PS:** Pinus sylvestris; **TB:** Taxus baccata; **Oryza sativa**: includes indica and japonica; **Alloteropsis spp.**: A. cimicina^d-g^, A. angusta^c,f,g,i^, A. semialata subsp semialata^b,f-i^, A. s. subsp eckloniana^g,i,j^; **Panicum spp.**: P. bisulcatum^n^, P. capillare^l^, P. coloratum^l^, P. fluviicola^l^, P. laetum^k,m^, P. miliaceum^k,l^, P. millegrana^o^, P. phragmitoides^l^, P. turgidum^l^.

^p^Soreng, *et al*.^56^.

^q^Chalupska, *et al*.^57^.
